# Early Life Exposure to Fructose Alters Maternal, Fetal and Neonatal Hepatic Gene Expression and Leads to Sex-Dependent Changes in Lipid Metabolism in Rat Offspring

**DOI:** 10.1371/journal.pone.0141962

**Published:** 2015-11-12

**Authors:** Zoe E. Clayton, Mark H. Vickers, Angelica Bernal, Cassandra Yap, Deborah M. Sloboda

**Affiliations:** 1 Liggins Institute and Gravida: National Centre for Growth and Development, University of Auckland, Aukland, New Zealand; 2 Departments of Biochemistry and Biomedical Sciences, McMaster University, Hamilton, Canada; 3 Department of Obstetrics and Gynaecology, McMaster University, Hamilton, Canada; 4 Department of Pediatrics, McMaster University, Hamilton, Canada; University of Arkansas for Medical Sciences, UNITED STATES

## Abstract

**Aim:**

Fructose consumption is associated with altered hepatic function and metabolic compromise and not surprisingly has become a focus for perinatal studies. We have previously shown that maternal fructose intake results in sex specific changes in fetal, placental and neonatal outcomes. In this follow-up study we investigated effects on maternal, fetal and neonatal hepatic fatty acid metabolism and immune modulation.

**Methods:**

Pregnant rats were randomised to either control (CON) or high-fructose (FR) diets. Fructose was given in solution and comprised 20% of total caloric intake. Blood and liver samples were collected at embryonic day 21 (E21) and postnatal day (P)10. Maternal liver samples were also collected at E21 and P10. Liver triglyceride and glycogen content was measured with standard assays. Hepatic gene expression was measured with qPCR.

**Results:**

Maternal fructose intake during pregnancy resulted in maternal hepatic ER stress, hepatocellular injury and increased levels of genes that favour lipogenesis. These changes were associated with a reduction in the NLRP3 inflammasome. Fetuses of mothers fed a high fructose diet displayed increased hepatic fructose transporter and reduced fructokinase mRNA levels and by 10 days of postnatal age, also have hepatic ER stress, and elevated IL1β mRNA levels. At P10, FR neonates demonstrated increased hepatic triglyceride content and particularly in males, associated changes in the expression of genes regulating beta oxidation and the NLRP3 inflammasome. Further, prenatal fructose results in sex-dependant changes in levels of key clock genes.

**Conclusions:**

Maternal fructose intake results in age and sex-specific alterations in maternal fetal and neonatal free fatty acid metabolism, which may be associated in disruptions in core clock gene machinery. How these changes are associated with hepatic inflammatory processes is still unclear, although suppression of the hepatic inflammasome, as least in mothers and male neonates may point to impaired immune sensing.

## Introduction

In recent decades, obesity has become a major public health issue, with two-thirds of adults in the United States classified as overweight or obese–proportions that are mirrored in Western countries and rising in developing countries world-wide [1, 2]. Obesity is one component of the metabolic syndrome, a combination of disorders, including insulin resistance, hypertriglyceridaemia and hypertension, which increase the risk of cardiovascular disease and Type 2 diabetes and contribute significantly to morbidity and mortality. Concomitant with increased obesity rates in the general population, the prevalence of obesity during pregnancy has doubled in the past two decades [3, 4]. Critically, maternal obesity has long term implications for the postnatal health status of her offspring [5]. It has been well-established that a mother’s nutritional status during pregnancy can permanently alter the physiology of her offspring; previous studies investigating maternal high-fat intake have shown that offspring exhibit a significantly increased risk of obesity and metabolic syndrome independent of their postnatal diet [6].

Coincident with the increase in prevalence of obesity has been an alarming increase in the consumption of fructose and high fructose corn syrup (HFCS), primarily in the form of soft-drinks, confectionary and processed foods. Fructose consumption has been demonstrated to be a significant contributing factor in the obesity epidemic [7, 8]. Short-term studies in humans, using fructose concentrations ranging from just 7.5% to 25% of caloric intake, have consistently demonstrated resultant hyperlipidaemia and insulin resistance [9–13]. Studies using animal models have shown that long-term fructose consumption leads to development of the metabolic syndrome in rodents, characterised by hypertension, hyperinsulinaemia, hypertriglyceridaemia, hepatic insulin resistance and non-alcoholic fatty liver disease (NAFLD). Even low levels of fructose intake (10%) have been shown to alter maternal leptin responsiveness and lead to impaired leptin signalling in the fetus [14]. In keeping with global nutritional trends, the effects of maternal intake of processed food sweeteners and fructose has received considerable attention [15–17].

Fructose is more lipogenic than glucose and consumption usually results in immediate elevations in plasma triglycerides. These lipogenic effects can be attributed to the metabolism of fructose in the liver, which is distinct from that of glucose. Fructose bypasses the rate-limiting step of glycolysis and uses a rapid ATP-requiring reaction that abruptly depletes ATP and provokes a compensatory rise in AMP [18]. Thus, fructose has the opposite effect of glucose on the AMPK/malonyl-CoA signaling system and therefore directly provides substrate for conversion to fatty acids and triglycerides without negative feedback as well as upregulating transcription factors and enzymes involved in lipogenesis, including sterol regulatory element binding protein 1c (SREBP1c) and Acetyl-CoA carboxylase (ACC). In addition, the intracellular ATP depletion that arises due to fructose intake also leads to generation of uric acid and potential for development of hyperuricemia [19]. Furthermore, fructose intake has been shown to reduce activity of hepatic PPARα in rats, leading to impaired fatty acid oxidation. Recent studies suggest these effects may be mediated through a Sirtuin- 1 (SIRT-1) dependent mechanism; in cultured hepatocytes, administration of fructose has been shown to lead to upregulation of lipogenic and gluconeogenic enzymes via SIRT[20].

Given the rise in prevalence of maternal obesity and the apparent obesogenic effects of a high-fructose diet, studies investigating the effects of maternal fructose intake on the offspring are relevant and necessary. Of note, the type of fructose utilised in experimental studies to date remains poorly described. Many studies have utilised high fructose corn syrup (HFCS) which is a commonly used sweetener in food and beverages. However, the terms HFCS and fructose are often, and incorrectly, used interchangeably. While pure crystalline fructose, as used in our previous work [21] and in the present study contains fructose alone, the most widely used variety of HFCS contains approximately 55% fructose and the rest as glucose. We developed a rat model of maternal fructose consumption, such that fructose comprised approximately 20% of total caloric intake, well within the range reported in human studies and representing a relevant caloric load from fructose. Using this model, we have previously shown that maternal fructose consumption leads to distinct sex-specific changes in placental growth and fetal and neonatal endocrine activity [21]. Based on these findings, we now present a follow-up study, which aimed to determine the effects of maternal fructose intake on lipid metabolism in the mother, as well as fetal and neonatal offspring, in particular the expression levels of key genes involved in carbohydrate, lipid and fatty acid metabolism.

## Materials and Methods

### Animal model

An animal model of maternal high-fructose intake was developed as previously described [21]. Virgin Wistar rats were time-mated using an estrus cycle monitor (EC-40, Fine Science Tools, Canada). Upon confirmation of mating via vaginal lavage, animals were housed individually and randomly assigned to either Control (CON) or Fructose (crystalline fructose, FR) groups. FR was administered from day 1 of pregnancy until postnatal day P(10) and was given at a concentration designed to provide an additional 20% of total daily calories from fructose. Body weight, food and water intakes of all animals were recorded daily and fructose concentrations adjusted daily according to changes in chow intake throughout gestation. All animals had ad-libitum access to a standard control chow diet (Diet 2018, Harlan, Oxon, UK). Three timepoints, embryonic day (E)21, postnatal day (P)2 and P10, were investigated. At E21, a subcohort of pregnant dams were sacrificed (N = 10 Cont and N = 9 FR) with isoflurane anaesthesia followed by decapitation. E21 fetuses were counted, weighed, sexed and position in uterine horn recorded, reabsorptions were counted and recorded. E21 fetuses were killed by decapitation, blood samples collected, and liver weights recorded. For those dams remaining in the trial, following spontaneous birth, litter size, birthweight and sex were recorded. At P2, litters were adjusted to 8 pups per litter (4 males and 4 females) to standardize nutrition. Remaining neonates were sacrificed using the same protocol as described for E21 fetuses and blood and liver samples were collected. The remaining dams (N = 8 CON and N = 5 FR) were killed at P10 and maternal and offspring blood and tissue samples weighed and collected. Maternal, fetal and neonatal bloods were centrifuged and plasma stored at -20C for later analysis. Maternal, fetal and neonatal liver samples were frozen in liquid nitrogen and stored at -80°C. All tissue collections occurred between 0900–1100 hours. All animal work was approved by the Animal Ethics Committee at the University of Auckland (Ethical Approval R712).

### Blood Biochemistry

All blood samples were centrifuged at 2500rpm at 4°C for 5 minutes (offspring) or 15 minutes (maternal) and supernatants collected and stored at -20°C until later analysis.. Plasma samples from E21 fetuses were pooled according to litter and sex in order to obtain sufficient plasma volume for all analyses. Plasma non-esterified free fatty acids (NEFA) concentrations were measured using a commercially available assay (Zen-Bio Inc, NC, USA, cat SFA-5) as per the manufacturer’s instructions. Blood biochemistry panels were performed using an automated biochemistry analyser (Hitachi 902 Autoanalyser, Roche Diagnostics, IN, USA) to obtain plasma levels of lipase, alkaline phosphatase concentrations (ALP), aspartate aminotransferase concentrations (AST), alanine transaminase concentrations (ALT) and total protein.

### Liver Triglyceride and Glycogen Content

Triglycerides were extracted from liver tissue using previously validated methods [22]. Frozen liver tissue (30mg) was homogenised in 2 ml of 2:1 chloroform-methanol and then incubated for 90 minutes on a rotating platform, after which 0.4 ml of methanol was added and the extract vortexed for 30 seconds before centrifugation at 2400g for 10 minutes at room temperature. The supernatant was pipetted into a new tube and 0.5 ml of 0.04% CaCl_2_ added before further centrifugation at 550 g for 20 minutes at room temperature. The upper phase was discarded and the interface between the upper and lower phases washed three times with 500μL of a chloroform-methanol-water (1.5 ml: 24 ml: 23.5 ml) wash. The final wash was removed and 0.05ml methanol was added before vortexing and freeze-drying the samples for one hour, using a centrifugal evaporator (HetoVac VR1 and CT110, Heto, Denmark). Extracts were then re-dissolved in 0.2 ml of 3:2 tert-butyl alcohol-Triton X-100 and stored at -20°C until triglyceride analysis. Immediately prior to analysis, samples were thawed and 100μL normal saline added. All samples were analysed in a single assay, using an automated bioanalyser (Roche/Hitachi GOD-PAP, Roche Diagnostics, Penzberg, Germany). Triglyceride levels are given in mmol/L.

Liver glycogen content was measured using a previously validated method [23]. Liver tissue (0.03g) was combined with 19 volumes (0.57ml) of 10mmol/L sodium acetate buffer (pH 4.6). Samples were homogenised on ice and 0.5ml homogenate transferred to a tube containing 0.1ml amyloglucosidase (60units/ml). The mixture was incubated at 37°C in a water bath for 2h to allow conversion of glycogen to free glucose. Samples were then centrifuged at 1000g for 5 minutes at room temperature and stored at -80°C. Glucose was analysed in an automated bioanalyser (Roche/Hitachi GOD-PAP, Roche Diagnostics, Penzberg, Germany), using the glucose oxidase method. The glucose generated in the reaction was used as a surrogate measure of liver glycogen content.

### Molecular Analyses

#### RNA extraction and reverse transcription (RT)

Total RNA was extracted from liver samples using a commercially available extraction kit (AllPrep Mini kit, Qiagen, cat 80204) as per the manufacturer’s instructions. Genomic DNA contamination was removed from each sample via treatment with RNase-free DNase (Invitrogen Life Technologies; Auckland, NZ) according to the manufacturer's instructions. RNA quantity and purity were analysed using a NanoDrop spectrophotometer (ND-1000; BioLab Ltd) using NanoDrop software (version 3.1.2). All RNA samples were stored at -80°C until required.

5μg of total RNA was used for first strand cDNA synthesis using a commercially available cDNA synthesis kit (Superscript® VILO™, Invitrogen, cat 11754–250) and a standard thermocycler (GeneAmp® PCR System 9700, Applied Biosystems, California, USA), under the following cycling conditions: an initial denaturation stage of 5 minutes at 96°C, followed by 30 cycles of 30 seconds each of 96°C (denaturation), 60°C (annealing stage) and 72°C (extension stage). cDNA was stored at -20°C.

#### Quantitative Polymerase Chain Reaction (qPCR)

For the quantification of hepatic gene expression levels and of the endogenous references cyclophilin and HPRT, a quantitative PCR assay was performed using the ABI PRISM® 7900HT Sequence Detection System (Applied Biosystems; Auckland, New Zealand). All primers were either designed using Primer 3 software (version 0.4.0, Whitehead Institute for Biomedical Research (Table 1) and manufactured by Invitrogen (Invitrogen Life Technologies; Auckland, New Zealand), or purchased from Qiagen (Table 1). Optimal primer conditions were adjusted to the following cycling conditions: Length: 20bp (range 17-23bp), Tm: 63°C (range 60–65°C), and amplicon length: 100-300bp. Dissociation analyses were performed to ensure specificity and samples producing a single peak in the dissociation curves were used. Amplified products were visualized on an agarose gel using the E-Gel® CloneWell 0.8% SYBR Safe gel (Invitrogen, cat G6618-08), run on the E-Gel® iBase™ Power System (Invitrogen, cat G6400) and sequenced by spectrophotometry (Allan Wilson Centre, Massey University). The resulting sequences were evaluated using NCBI BLAST to ensure specificity.

**Table 1:** Primer Sequences and Qiagen QuantiTect Primer Assays™.

Rat Gene	Forward Primer	Reverse Primer	Amplicon Length (bp)	GenBank Accession Number
*HPRT*	GGTCCTTTTCACCAGCAAGCT	TGACACTGGCAAAACAATGCA	195	NM_012583
*Cyclophilin a*	TTGGGTCGCGTCTGCTTCGA	GCCAGGACCTGTATGCTTCA	240	NM_017101.1
*BMAL1*	ACTGCACCTCGGGAGCGACT	CGCCCGATTGCAACGAGGCA	320	NM_024352.2
*CLOCK*	ACCGCACCTGCCAGCTCATG	GCGTGTCCGCTGCTCTAGCT	214	NM_021856.1
*CRY1*	CGGAAGCTCGTGTCGGTCCG	CGCGCGACGTCCTTCAGGAG	232	NM_198750.2
*CRY2*	ACGGTCCCCGTGCAGTCGAT	CTGACGAGGAGGCCGCGAAC	166	NM_113405
*XBP1s*	GAGTCCGCAGCAGGTG	GCGTCAGAATCCATGGGA	165	NM_001004210
*XBP1t*	GAGCAGCAAGTGGTGGATTT	TCTCAATCACAAGCCCATGA	197	NM_001004210
*GRP78*	CCACCTATTCCTGCGTCGG	CAATCAGACGCTCCCCTTCA		NM_013083.2
*SREBP1c*	AGCCGTGGTGAGAAGCGCAC	TGAGGGTGGAGGGGTCAGCG	300	NM_001276707.1
**Qiagen QuantiTect Primer Assays™**				
**Rat Gene**	**Catalogue Number**			**GenBank Accession Number**
*PER1*	QT01615726			NM_001034125
*PER2*	QT00184737			NM_031678
*SIRT1*	QT02345854			NM_144759
*NFκB (RelA)*	QT01580012			NM_199267
*IL-1β*	QT00181657			NM_031512
*NLRP3*	QT01568448			NM_001191642
*NFKBIA*	QT01600956			NM_001105720
*GLUT5 (Slc2a5)*	QT00177821			NM_031741

**Abbreviations:** HPRT, Hypoxanthine-guanine phosphoribosyltransferase; BMAL1, brain and muscle arnt-like protein 1; Clock, circadian locomotor output cycles kaput; Cry1 and Cry2, cryptochrome 1 & 2; XBP1s/ XBP1t, x-box binding protein spliced & total; GRP78, 78 kDa glucose-regulated protein or Binding immunoglobulin protein(BiP); SREBP1c, Sterol Regulatory Element-Binding Protein 1c; PER1 and PER2, period 1 & 2 genes; SIRT1, sirtuin 1; *NFκB (RelA)*, nuclear factor kappa-light-chain-enhancer of activated B cells; IL-1β, interleukin 1-beta; NLRP3, NLR family, pyrin domain containing 3, NFKBIA, nuclear factor of kappa light polypeptide gene enhancer in B-cells inhibitor alpha; GLUT5, facilitated glucose/fructose transporter member 5.

Quantification of gene expression levels were performed under the following conditions: an initial 2 min hold period at 50°C for normalisation (Stage 1), followed by enzyme activation at 95°C for 2 min (Stage 2); amplification of the gene product through 40 successive cycles of 95°C for 15 sec then 60°C for 1 min (Stage 3); followed by a dissociation stage of 15 sec at 95°C, 15 sec at 60°C and 2 min at 99°C (Stage 4). A standard curve was generated from the mean cycle threshold (Ct) of eight standards (1:5 serial dilution) of a known concentration in triplicate, while amplification and dissociation curves were generated for all standards and samples (Applied Biosystems, California, USA). Each sample was run in triplicate.

#### Fatty acid metabolism PCR Array

Expression levels of 84 genes involved in hepatic fatty acid metabolism were measured using a commercially available RT^2^ *Profiler* PCR array. Rat Fatty Acid Metabolism PCR Array, (cat PARN-007, Format G, Qiagen Sciences, Maryland 20974, USA) and RT^2^ Real-Time SyBR Green/ROX PCR Mix were purchased from Qiagen (Frederick, MD). PCR was performed on Roche LightCycler® 480 II Real-Time PCR System (Roche Diagnostics, Roche Applied Science, Penzberg, Germany) according to the manufacturer's instructions. Samples from four P10 control and four P10 neonates born to FR-fed mothers were compared. For data analysis, the ΔΔCt method was used with the aid of a Microsoft excel spreadsheet containing algorithms provided by the manufacturer (http://www.sabiosciences.com/pcrarraydataanalysis.php) and fold-changes were calculated.

### Statistical Analyses

Unless otherwise stated, maternal data were analyzed by t-test, and offspring data were analyzed by two-way factorial ANOVA, with maternal diet and offspring sex as factors. In all cases, data that were not normally distributed were log transformed to achieve data normality. Post-ANOVA comparisons among means were made using Tukey’s post hoc for multiple comparisons. A p-value of < 0.05 was considered statistically significant.. All statistical evaluation was performed using GraphPad Prism version 6 for Windows (GraphPad Software Inc, La Jolla, CA, USA). As stated above, data from the RT^2^ *Profiler* PCR array was analyzed using the RT^2^ Profiler PCR Array web-based software (version 3.4, Qiagen, Maryland 20874, USA) and presented as fold change from control. All other data are presented as mean ± SEM, unless otherwise stated.

## Results

### Maternal Outcomes

We have previously reported that maternal fructose intake did not change absolute maternal body weight during gestation and lactation to P10 [21]. There were no differences in fat mass or lean mass between CON and FR dams at either E21 or P10, although FR dams had significantly higher liver weights when adjusted for body weight. There was no effect of maternal diet on fetal or neonatal body weight or growth to P10 [21], but circulating maternal fructose levels were higher as a result of prenatal fructose intake [21]. FR dams demonstrated increased total caloric intake and maternal hyperinsulinaemia at E21. FR intake did not alter maternal blood glucose, β-hydroxybutyrate (BHB), or electrolyte levels at either time point [21].

#### Prenatal fructose intake significantly alters gene expression of maternal hepatic fructose and glucose metabolic enzymes

**Fig 1:**
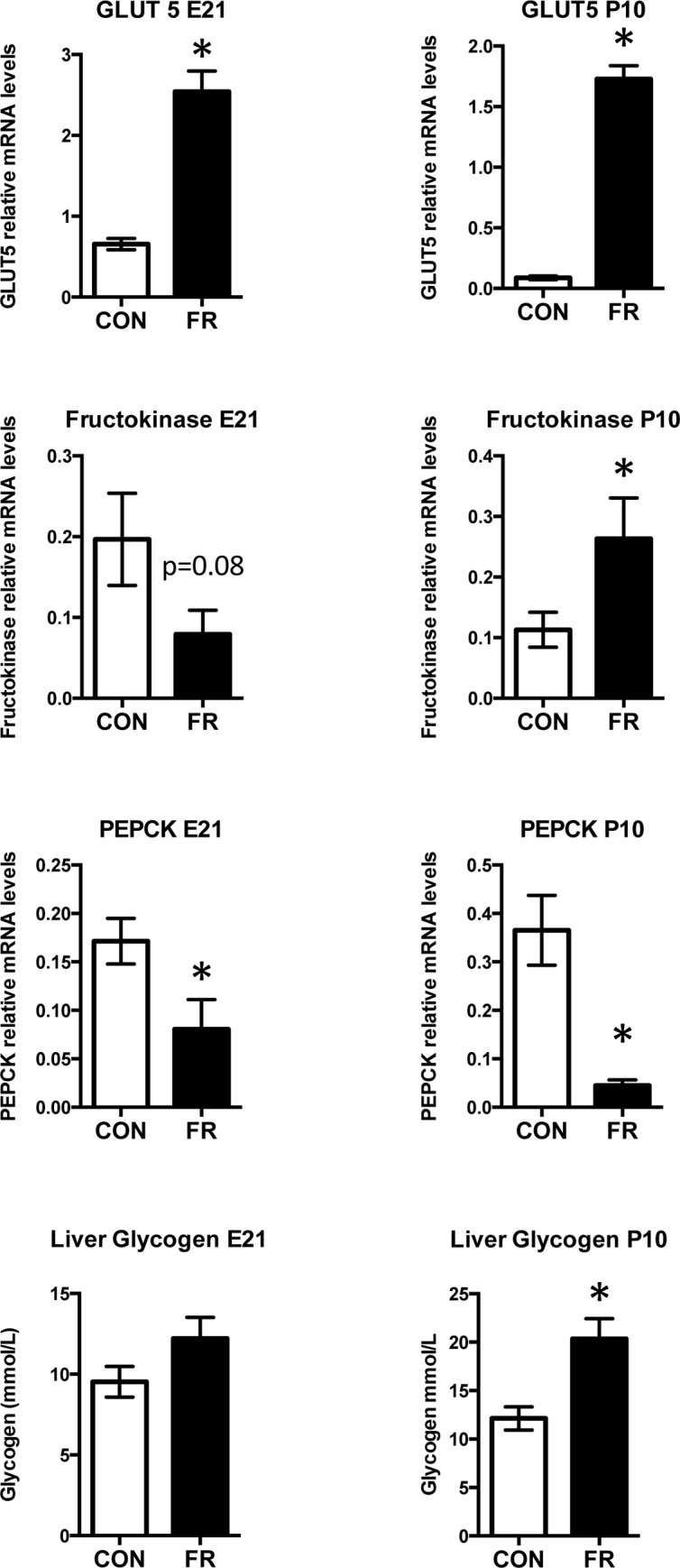
Prenatal fructose intake significantly alters gene expression of maternal hepatic fructose and glucose metabolic enzymes. Maternal GLUT5, fructokinase, PEPCK mRNA and liver glycogen levels at 2 timepoints. Data are presented as means ± S.E.M. All mRNA levels are relative to the geometric mean of housekeeping genes. * p < 0.05 compared to control fed mothers; n = 10 CON, n = 9 FR. CON: Control fed mothers, FR: Fructose fed mothers. E21: embryonic day 21, P10: postnatal day 10. GLUT5: fructose transporter, PEPCK: phosphoenolpyruvate carboxykinase.

Fructose intake during pregnancy significantly elevated the fructose specific transporter GLUT5 mRNA levels at both E21 (p<0.0001) and P10 (p<0.0001) timepoints (Fig 1). Fructokinase mRNA levels were lower at E21 (p = 0.08) but were significantly higher at P10 (p = 0.04) (Fig 1). These changes were accompanied by a suppression in the rate limiting enzyme involved in gluconeogenesis, phosphoenolpyruvate carboxykinase (PEPCK) at both E21 and P10 time points (p<0.05) (Fig 1). Since a portion of ingested fructose is converted to glycogen, we examined maternal liver glycogen levels. Prenatal fructose intake increased maternal liver glycogen levels and although levels at E21 were not statistically significant (p = 0.1) at P10 maternal liver glycogen was significantly elevated in FR mothers (p = 0.003) (Fig 1).

#### Prenatal fructose intake significantly alters lipid metabolism in maternal liver

**Fig 2:**
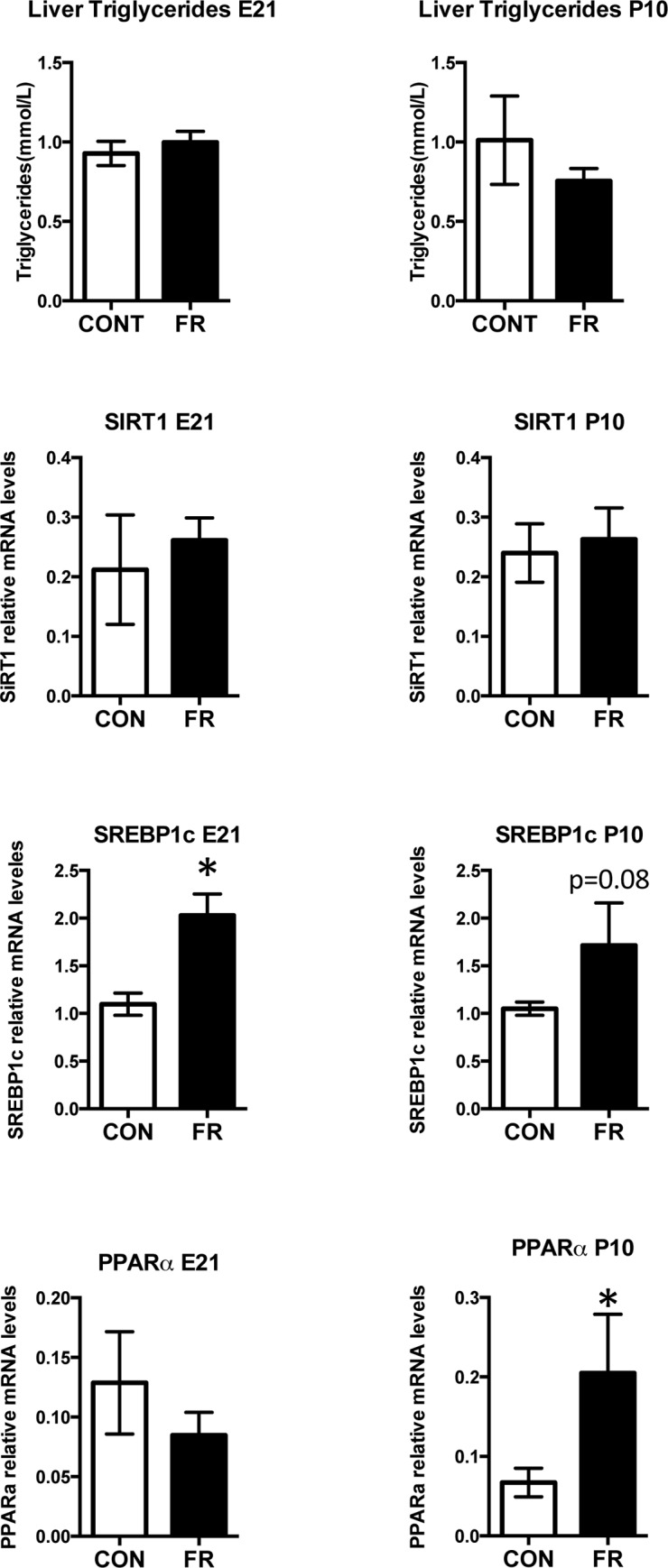
Prenatal fructose intake significantly alters lipid metabolism in maternal liver. Maternal SIRT1, SREBP1c, PPARa, and liver triglyceride levels at 2 timepoints. Data are presented as means ± S.E.M. All mRNA levels are relative to the geometric mean of housekeeping genes. * p < 0.05 compared to control fed mothers; n = 10 CON, n = 9 FR. CON: Control fed mothers, FR: Fructose fed mothers. E21: embryonic day 21, P10: postnatal day 10. SIRT1: Sirtuin 1, SREBP1c: sterol regulatory element-binding protein 1-c, PPARa: peroxisome proliferator-activated receptor alpha.

In the liver, fructose provides substrates for glycolysis, glycogenesis as well as lipogenesis and fatty acid esterification [24, 25]. We thus investigated maternal hepatic lipogenesis and triglyceride deposition. Although prenatal fructose intake did not significantly alter maternal liver triglyceride levels (Fig 2), circulating maternal NEFA was significantly lower in FR mothers at P10 (Table 2). Maternal plasma lipase was decreased in FR mothers at E21 (p = 0.002) but unchanged at P10 (Table 2). Although plasma ALP was similar between groups, prenatal fructose intake decreased both AST (p = 0.01) and ALT (p = 0.02) at E21 (Table 2). At P10 however, both AST (p = 0.01) and ALT (p = 0.02) levels were significantly elevated in FR mothers compared to control (Table 2).

**Table 2:** Maternal blood biochemistry.

	****E21****			****P10****		
	Control	Fructose	P value	Control	Fructose	P value
NEFA (uM)	7086 ± 830	6713 ± 849	>0.05	2617 ± 302	1553 ± 207*	0.028
Lipase (IU)	66 ± 3.4	40 ± 6.4*	0.0026	73.1 ± 2.7	78 ± 2.9	0.25
ALP (IU)	2.7 ± 0.2	2.4 ± 0.2	0.36	6.2 ± 1.3	6.8 ± 1.1	0.76
AST (IU)	594 ± 127.1	220 ± 34.6*	0.01	58.1 ± 10	210.7 ± 76*	0.01
ALT (IU)	207 ± 73.5	22.3 ± 2.9*	0.02	49 ± 4.3	82.1 ± 14.5*	0.02
Total protein (IU)	0.32 ± 0.01	0.27 ± 0.05	0.42	0.24 ± 0.03	0.33 ± 0.04	0.17

Data are presented as mean ± SEM.

* p<0.05 compared to control. NEFA (uM): non-esterified fatty acid; ALP: Alkaline phosphatase; AST: Aspartate aminotransferase; ALT: alanine aminotransferase.

Maternal hepatic sterol regulatory element-binding protein 1-c (SREBP-1c) mRNA levels were increased at E21 (p = 0.002) and modestly higher at P10 (p = 0.08) (Fig 2). Although peroxisome proliferator-activated receptor alpha (PPARα) mRNA levels were similar at E21, FR mothers had elevated levels at P10 (p = 0.04) (Fig 2).

#### Prenatal fructose intake induces maternal hepatic endoplasmic reticulum (ER) stress but does not induce hepatic inflammasome-induced pro-inflammatory signalling

**Fig 3:**
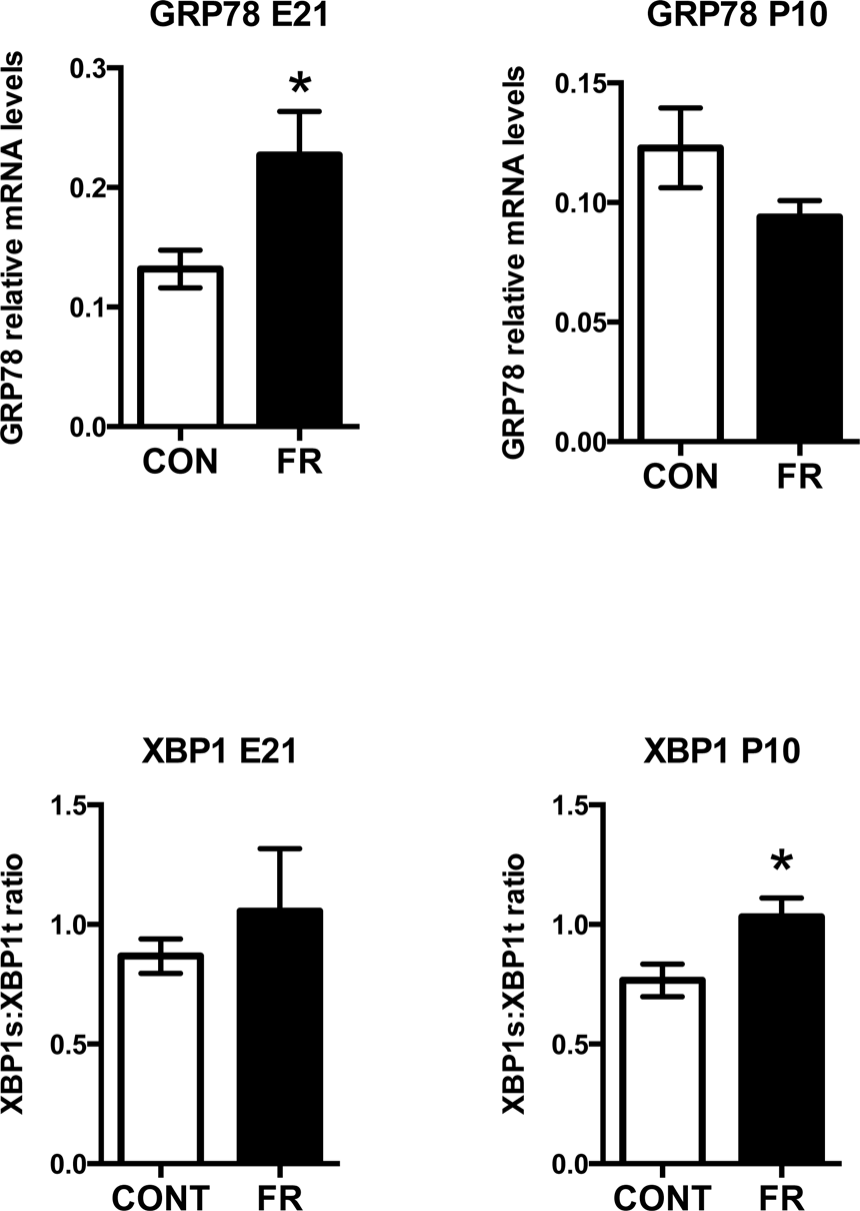
Prenatal fructose intake induces maternal hepatic endoplasmic reticulum (ER) stress. Maternal GRP78 and XBP1 mRNA levels at 2 timepoints. XBP1 is expressed as a ratio of spliced to total mRNA levels. Data are presented as means ± S.E.M. All mRNA levels are relative to the geometric mean of housekeeping genes. * p < 0.05 compared to control fed mothers; n = 10 CON, n = 9 FR. CON: Control fed mothers, FR: Fructose fed mothers. E21: embryonic day 21, P10: postnatal day 10. GRP78: 78 kDa glucose-regulated protein, XBP1: X-box binding protein 1.

**Fig 4:**
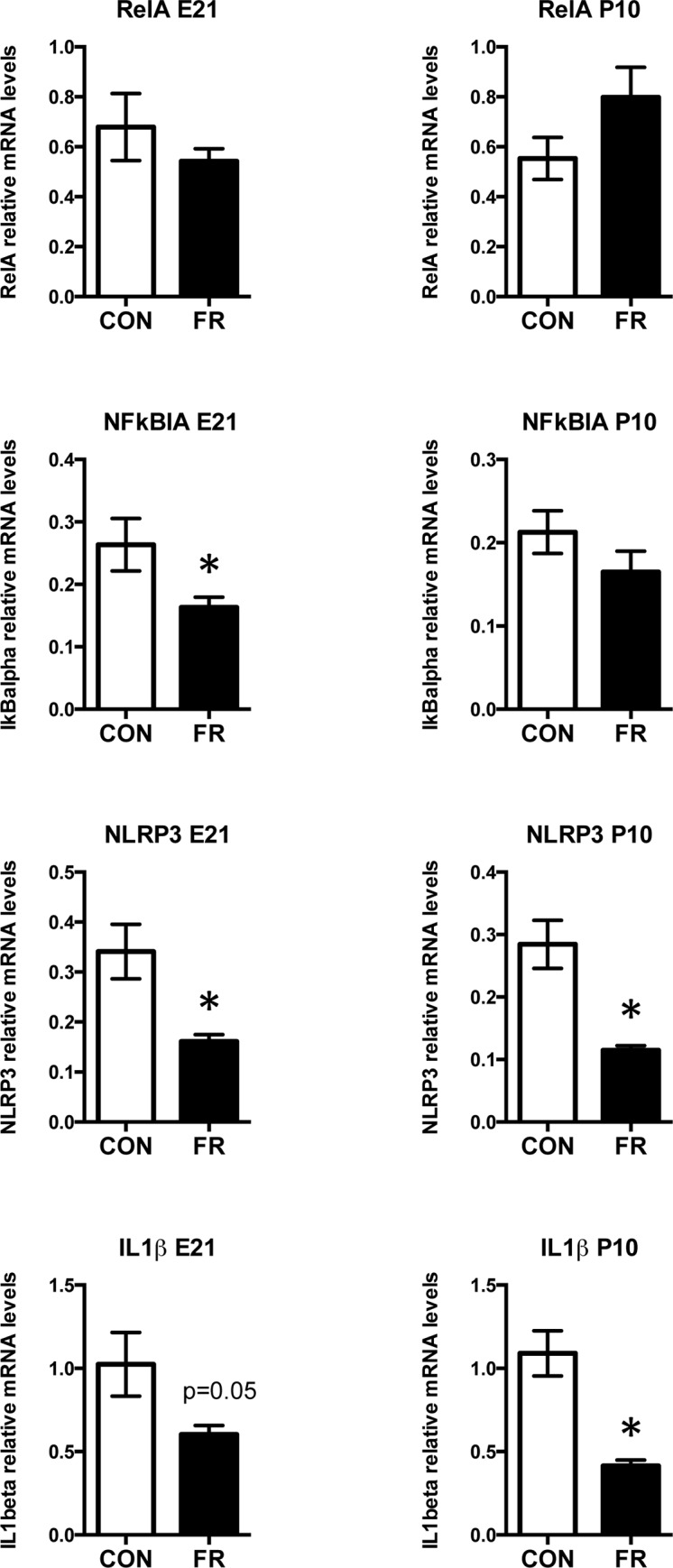
Maternal hepatic NLRP3 inflammasome and inflammatory signalling. Maternal RelA (NFκB), NFκBIA, NLRP3 inflammasome and IL-1β mRNA levels at 2 timepoints. Data are presented as means ± S.E.M. All mRNA levels are relative to the geometric mean of housekeeping genes. * p < 0.05 compared to control fed mothers; n = 10 CON, n = 9 FR. CON: Control fed mothers, FR: Fructose fed mothers. E21: embryonic day 21, P10: postnatal day 10. RelA: gene encoding NFκB—nuclear factor NF-kappa-B p65 subunit, NFκBIA: nuclear factor of kappa light polypeptide gene enhancer in B-cells inhibitor, NLRP3: NOD-like receptor family, pyrin domain containing 3, IL1β: interleukin 1 beta.

Fructose has been shown to induce ER stress responses [26] and in this manner thought to contribute to changes in hepatic lipogenesis and inflammation [27] potentially through inflammasome induction [28]. We thus investigated maternal markers of ER stress and inflammasome-induced changes in pro-inflammatory cytokine mRNA levels after prenatal fructose ingestion. Fructose intake during pregnancy significantly elevated mRNA levels of the ER chaperone and signaling regulator GRP78 at E21 (p<0.001) but not at P10 (Fig 3), and elevated a critical transcription factor, XBP1 in maternal livers. XBP1 splicing is induced by ER stress and the ratio of spliced to total XBP1 (XBP1s:XBP1t) is used as a marker of ER stress [29]. Maternal fructose intake induced an increase in maternal liver XBP1s:XBP1t mRNA ratio, although the increase was significant only at P10 (p<0.01) (Fig 3). Much to our surprise, increased markers of ER stress were not accompanied by an increase in the mRNA levels of NRLP3. In fact, prenatal maternal fructose induced a decrease in NLRP3 mRNA levels at both E21 and P10 and an associated decrease in ILβ at both timepoints (Fig 4).

### Offspring outcomes

We have previously reported a small but significant increase in birthweights in FR offspring compared to control, with associated increases in circulating fructose, insulin and leptin levels in female, but not male, fetuses and elevated plasma fructose levels in all fructose-exposed neonates at P10 [21]. In the present follow-up study, we show that circulating levels of non-esterified free fatty acid (NEFA) levels, tended to be higher in female FR fetuses than in controls, but this difference did not reach statistical significance (main effect of diet p = 0.08, effect of sex p >0.05, no interaction) (Table 3). Lipase and AST levels were similar between groups and between sexes at both E21 and P10, although an interaction between diet and sex was observed in fetal plasma ALT levels (Table 3). Although fructose main effects were not observed, we show a significant sex effect and interaction between diet x sex on fetal plasma ALP levels (effect of sex p = 0.03, interaction p < 0.01). Tukey’s post hoc analysis revealed that fructose exposed male fetuses had higher ALP compared to control, and although fructose exposed females also had higher levels of ALP compared to controls, this difference did not reach statistical significance (Table 3). ALP values were similar between groups at P10. Prenatal fructose exposure suppressed total protein levels (main effect of diet p < 0.01, effect of sex p = 0.02, no interaction). Tukey’s post hoc analysis revealed a significant effect of sex on protein levels where female fetuses had higher protein levels compared to males (Table 3).

**Table 3:** Offspring blood biochemistry.

	Embryonic day 21					Postnatal day 10				
	Control		Fructose			Control		Fructose		
	Male	Female	Male	Female	Main effects	Male	Female	Male	Female	Main effects
**NEFA**	380 ± 25	367 ± 18	401 ± 24	451 ± 39	diet p = 0.08	5887 ± 366	6753 ± 378	6867 ± 453	5770 ± 383	diet NS
**(uM)**					sex NS					sex NS
					inter’n NS					inter’n p = 0.03
**Lipase (IU)**	62.1 ± 15	68.1 ± 5.1	75.4 ± 6.8	75.2 ± 7.6	NS	83.1 ± 25	69.6 ± 5.8	71.8 ± 3.3	64 ± 3.3	NS
**ALP**	2.23 ± 0.36^a^	0.66 ± 0.19^b^	0.85 ± 0.06^b^	1.32 ± 0.23^ab^	diet NS,	18.6 ± 2.0	16.51 ± 1.46	19.9 ± 2.3	22.9 ± 2.3	NS
**(IU)**					sex p = 0.03					
					inter’n p<0.01					
**AST (IU)**	4.68 ± 1.16	1.64 ± 0.55	4.53 ± 1.31	2.63 ± 0.44	NS	531 ± 170	485 ± 125	393 ± 116	630 ± 214	NS
**ALT**	33.3 ± 3.3	22.1 ± 2.0	24.8 ± 1.6	31.7 ± 3.7	diet NS	154.3 ± 14.9	158 ± 15.2	157.5 ± 48.8	178.7 ± 50.6	NS
**(IU)**					sex NS					
					inter’n p<0.01					
**Protein**	0.19 ± 0.04^a^	0.14 ± 0.02^a^	0.38 ± 0.05^b^	0.26 ± 0.02^ab^	diet p<0.01	0.22 ± 0.05	0.22 ± 0.04	0.30 ± 0.02	0.26 ± 0.02	NS
**(IU)**					sex p = 0.02					
					inter’n NS					

Data are presented as mean ± SEM. Values with different letters indicates significant differences. NS: not significant; inter’n: interaction; NEFA: non-esterified fatty acid; ALP: Alkaline phosphatase; AST: Aspartate aminotransferase; ALT: alanine aminotransferase, protein: total protein.

#### Prenatal fructose exposure increases fetal and neonatal hepatic fructose transporter levels

**Fig 5:**
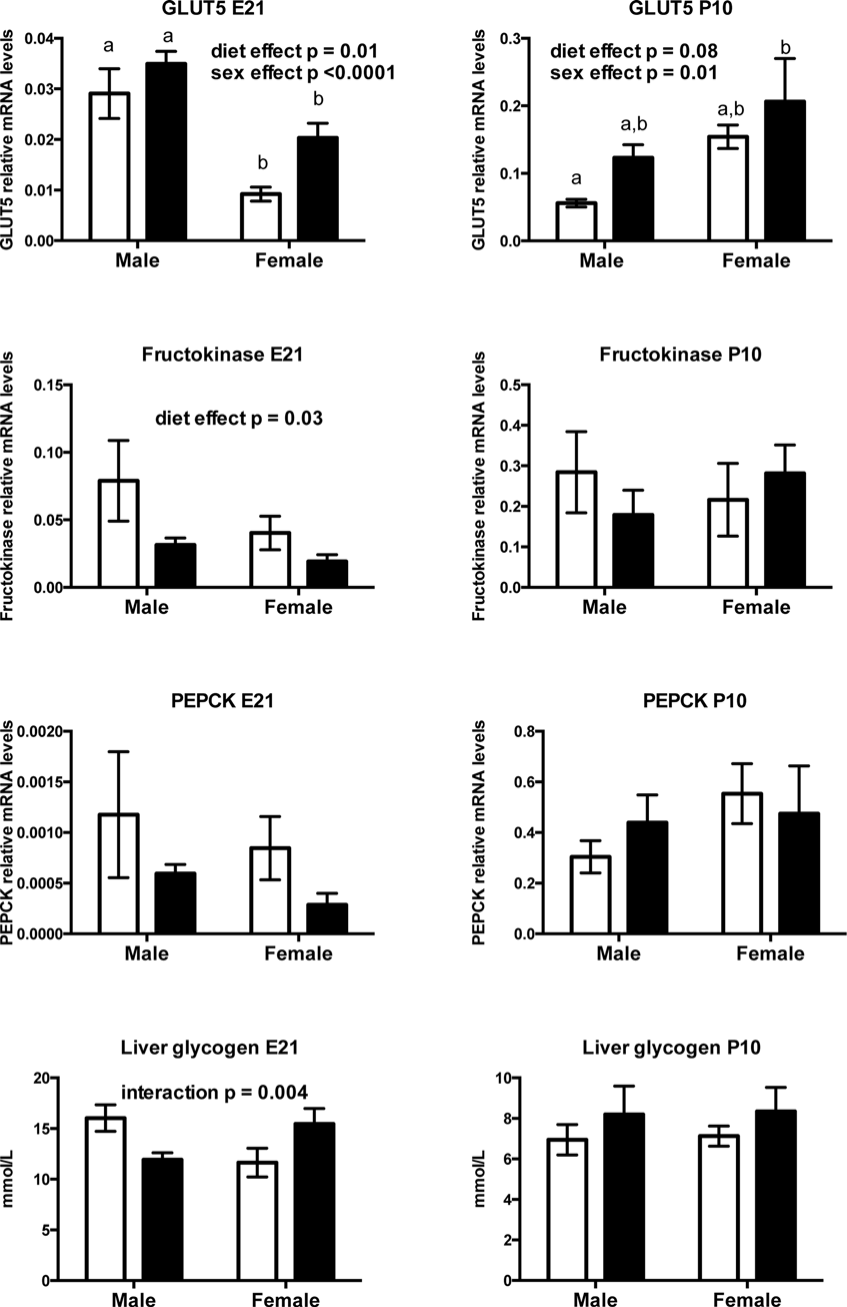
Prenatal fructose exposure increases fructose transport and decreases fructose metabolism in offspring. Offspring GLUT5, fructokinase, PEPCK mRNA and liver glycogen levels at 2 timepoints. Data are presented as means ± S.E.M. All mRNA levels are relative to the geometric mean of housekeeping genes. Two-Way ANOVA Main Effects are indicated in text where the 2 factors are maternal diet (fructose) and offspring sex. Tukey’s *post-hoc* analyses are indicated by letters, where bars with different letters indicate significance p < 0.05. Control offspring are in open bars, fructose exposed offspring are in black bars. E21: embryonic day 21, P10: postnatal day 10. n = 6 per group per sex.

Prenatal fructose exposure significantly elevated fructose specific transporter GLUT5 mRNA levels in fetal livers at E21 (Main effect of diet p = 0.013, effect of sex p < 0.001, no interaction) and modestly at P10 in a sex-dependant manner (Main effect of diet p = 0.08, effect of sex p = 0.01, no interaction) (Fig 5). Tukey’s *post-hoc* analysis at E21 demonstrated increased levels of GLUT5 mRNA in Cont and FR males compared to Cont and FR females (Cont male versus Cont and FR female p<0.05; FR male versus Cont and FR female p<0.05) (Fig 5). At P10, Tukey’s *post hoc* analysis demonstrated that FR females had the highest levels of GLUT5 mRNA, although only reached significant difference compared to Cont males (p<0.05) (Fig 5).

Hepatic fructokinase mRNA levels were lower at E21 (Main effect of diet p = 0.03, effect of sex p = 0.13, no interaction) but were not different at P10 (Main effect of diet p = 0.8, effect of sex p = 0.8, no interaction) (Fig 5). Although PEPCK was lower at E21, this difference was not significant and no overall effect of sex or diet x sex interaction was observed (Fig 5). PEPCK mRNA levels at P10 were also not affected by prenatal fructose intake. Hepatic glycogen levels were similar between Cont and fructose exposed fetuses at E21 (p = 0.9) and neonates at P10 (p = 0.7) and demonstrated no effect of sex at either timepoint, although we observed a diet x sex interaction (p = 0.004) where fructose exposure decreased liver glycogen in males but increased liver glycogen in females (Fig 5). Liver glycogen levels were similar between groups and between sexes at P10 (Fig 5).

#### Prenatal fructose exposure increases hepatic lipid content, and modifies transcription factors and fatty acid oxidative enzymes in neonates in a sex-dependent manner

**Fig 6:**
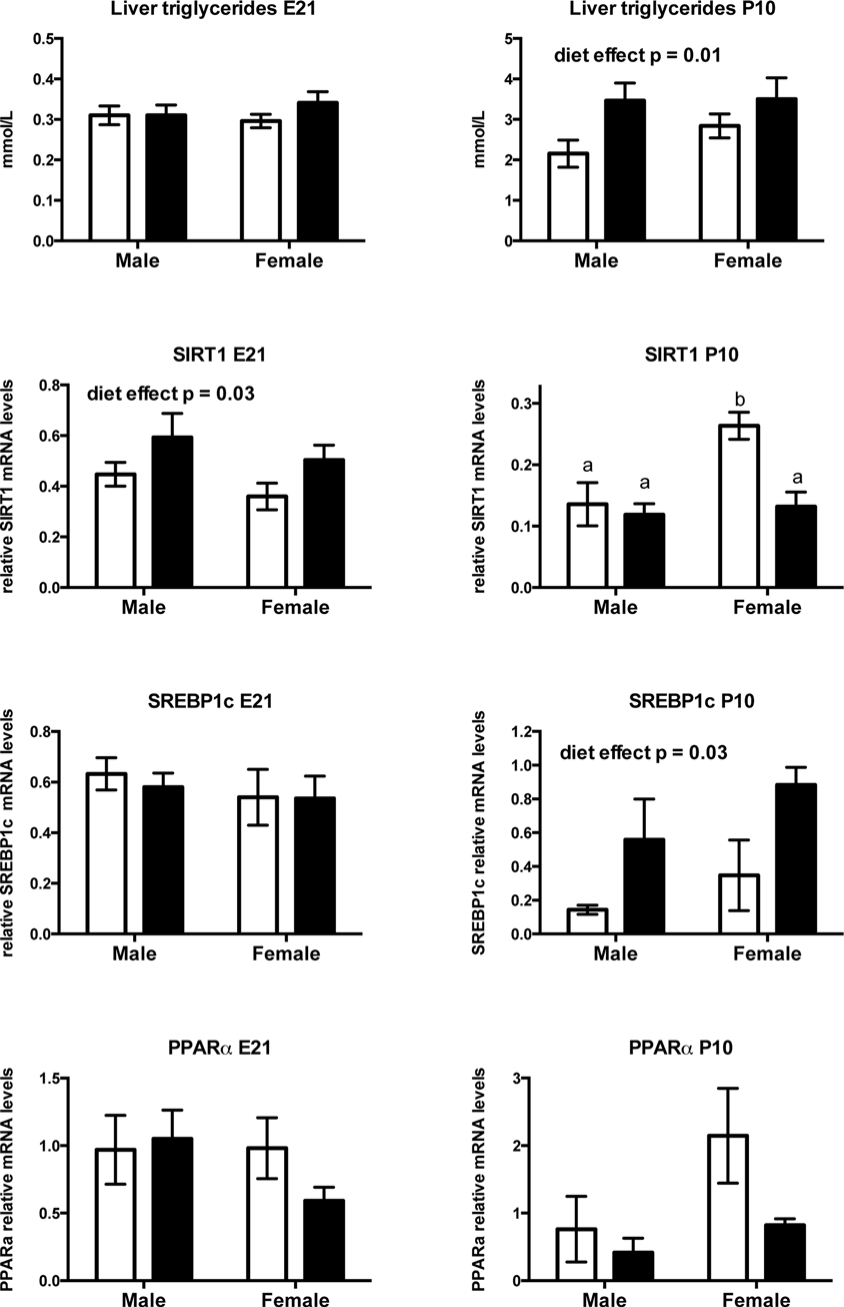
Prenatal fructose exposure increases hepatic lipid content, and modifies transcription factors and fatty acid oxidative enzymes in neonates in a sex dependent manner. Offspring liver triglyceride levels and SIRT1, SREBP1c, PPARa, mRNA levels at 2 timepoints. Data are presented as means ± S.E.M. All mRNA levels are relative to the geometric mean of housekeeping genes. Two-Way ANOVA Main Effects are indicated in text where the 2 factors are maternal diet (fructose) and offspring sex. Tukey’s *post-hoc* analyses are indicated by letters, where bars with different letters indicate significance p < 0.05. Control offspring are in open bars, fructose exposed offspring are in black bars. E21: embryonic day 21, P10: postnatal day 10. n = 6 per group per sex.

Fetal liver triglyceride content was similar between groups and between sexes at E21, but by P10 fructose-exposed neonates demonstrated higher hepatic triglyceride levels (main effect of diet p = 0.019, effect of sex p = 0.36, no interaction) (Fig 6). Since dysregulation of hepatic fatty acid synthesis enzymes leads to hepatic lipid deposition, we investigated key transcriptional regulators in hepatic lipid metabolism. Sirtuin 1 lies upstream of two key transcriptional regulators, SREBP and PPARa to regulate fatty acid metabolism. Prenatal fructose exposure resulted in a significant increase in mRNA levels of SIRT1 at E21 regardless of sex (main effect of diet p = 0.03, effect of sex p = 0.18, no interaction) (Fig 6). In neonates at P10 fructose exposure decreased SIRT1 mRNA levels in a sex dependent manner (effect of diet p = 0.009, effect of sex p = 0.01, diet x sex interaction p = 0.03). Tukey’s post hoc analysis revealed that females at P10 were most vulnerable to prenatal fructose exposure showing a significant decrease in SIRT1 mRNA levels compared to all other groups. Although mRNA levels of SREBP1c were similar across all groups at E21, prenatal fructose exposure significantly increased levels in neonates at P10 regardless of sex (main effect of diet p = 0.03, effect of sex p = 0.2, no interaction) (Fig 6). PPARa mRNA at E21 and P10 were unchanged by prenatal fructose exposure (Fig 6).

#### Neonatal Hepatic Fatty Acid Metabolism

**Fig 7:**
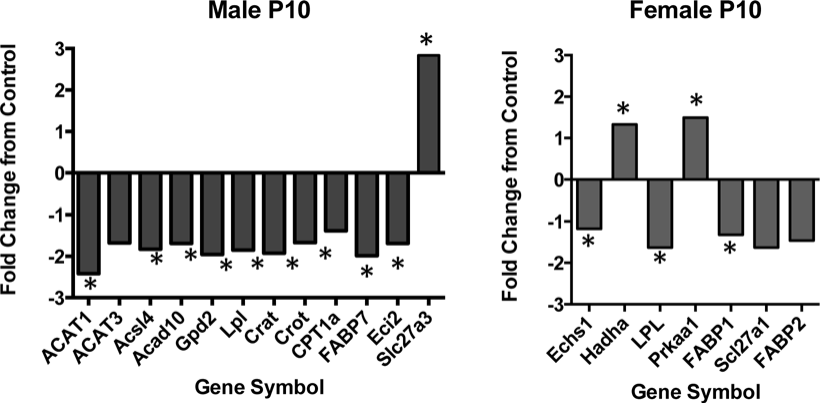
Hepatic gene expression levels of enzymes involved in free fatty acid metabolism in neonates. Neonatal P10 liver samples were analyzed using an RT^2^ profiler apoptosis PCR array. Data are presented as the fold difference from control, calculated from average ΔCT normalized to housekeeping genes. * p < 0.05 compared to control fed mothers, n = 4 per group, per sex.

To investigate further the impact of prenatal fructose exposure on fatty acid metabolism, we utilized a commercially available PCR array that investigates up to 84 genes involved in free fatty acid metabolism to uncover novel changes in enzymatic pathways. Since we observed the greatest changes in fatty acid regulation at P10, only neonatal P10 liver samples were investigated using this method. In males, the majority of the genes that were statistically different from control were downregulated (Fig 7). Many of these downregulated genes encode enzymes involved in the β oxidation of free fatty acids (Table 4, Fig 7) including ACAT1 (p = 0.03), Acsl4 (p = 0.017), Acad10 (p = 0.03), Crat (p = 0.006), Crot (p = 0.016), FABP7 (p = 0.05), and Eci2 (p = 0.04) which demonstrated mRNA levels that were significantly lower in FR male neonates compared to controls. Other genes (ACAT3 p = 0.089, CPT1a p = 0.07) were similarly decreased but differences did not reach statistical significance (Fig 7) (Table 4). In males, Slc27a3, the gene encoding the long chain fatty acid transport protein 3, was significantly upregulated (2.83 fold p = 0.03, Fig 7) in FR male neonates compared to control.

**Table 4:** Free fatty acid metabolic genes regulated by prenatal fructose exposure in male neonatal livers.

*Gene*	*Protein encoded*	*Function*	*Fold change*	*p value*
**ACAT1**	Acetyl-coA acetyltransferase	Ketone metabolism	-2.42	0.03
**ACAT3**	Acetyl-coA acetyltransferase	Ketone metabolism	-1.68	0.089
**Acsl4/ Facl4**	Long chain fatty acyl-CoA synthetase	Breakdown of complex fatty acids	-1.83	0.017
**Crat**	Carnitine acyltransferase	Beta oxidation	-1.93	0.006
**Crot**	Carnitine acyltransferase	Beta oxidation	-1.6	0.017
**CPT1a**	Carnitine acyltransferase	Beta oxidation	-1.38	0.07
**Fabp7**	Fatty acid binding protein 7	Fatty acid transport	-1.95	0.01
**Gpd2**	Glycerol-3-Phosphate dehydrogenase	glycerol phosphate shuttle and redox potential	-1.96	0.03
**Lpl**	Lipoprotein Lipase	Rate limiting enzyme in triglyceride hydrolysis	-1.86	0.02
**Eci1**	Enoyl-CoA isomerase	Metabolism of unsaturated fatty acids	-1.69	0.04
**Acad10**	Acyl-Coa dehydrogenase	Rate limiting step in beta oxidation	-1.69	0.03
**Slc27a3**	Long chain fatty acid Transport protein 3	Fatty Acid Transport	2.8	0.03

Data are presented as fold change from control values. (-) indicates a decrease in gene expression.

In females, genes involved in free fatty acid metabolism were differentially regulated. Levels of fatty acid binding proteins (FABP1 p = 0.003; FABP2 p = 0.06, Scl27a1 p = 0.07) and enoyl coenzyme a hydratase (ECHS1, p = 0.02) were reduced and mRNA levels of hadha (Hydroxyacyl-Coenzyme A dehydrogenase) and protein kinase aa1 (PRKaa1; AMPK) were significantly increased in female FR neonates compared to CON (Table 5).

**Table 5:** Free fatty acid metabolic genes regulated by prenatal fructose exposure in female neonatal livers.

*Gene*	*Protein encoded*	*Function*	*Fold change*	*p value*
**Echs1**	Enoyl Coenzyme A hydratase, short chain 1	Beta oxidation	-1.17	0.02
**Fabp1**	Fatty acid binding Protein 1	Fatty acid transport	-1.32	0.003
**Fabp2**	Fatty acid binding protein 2	Fatty acid transport	-1.5	0.06
**Lpl**	Lipoprotein Lipase	Rate limiting enzyme in triglyceride hydrolysis	-1.6	0.02
**Slc27a1**	Long chain fatty acid Transport protein 1	Fatty Acid Transport	-1.6	0.07
**Hadha**	Hydroxyacyl-Coenzyme A dehydrogenase	Mitochondrial trifunctional enzyme: beta oxidation	1.33	0.007
**Prkaa1**	AMPK	Metabolic master regulator	1.5	0.003

Data are presented as fold change from control values. (-) indicates a decrease in gene expression.

Interestingly, only one gene was altered in both female and male neonates at P10. Prenatal FR exposure resulted in a significant decrease in mRNA levels of lipoprotein lipase (LPL) in both male (1.85 fold decrease, p = 0.02) and female (1.6 fold decrease, p = 0.02) neonates compared to CON offspring (Fig 7).

#### Prenatal fructose intake induces neonatal, but not fetal hepatic endoplasmic reticulum (ER) stress and induces pro-inflammatory cytokine expression

**Fig 8:**
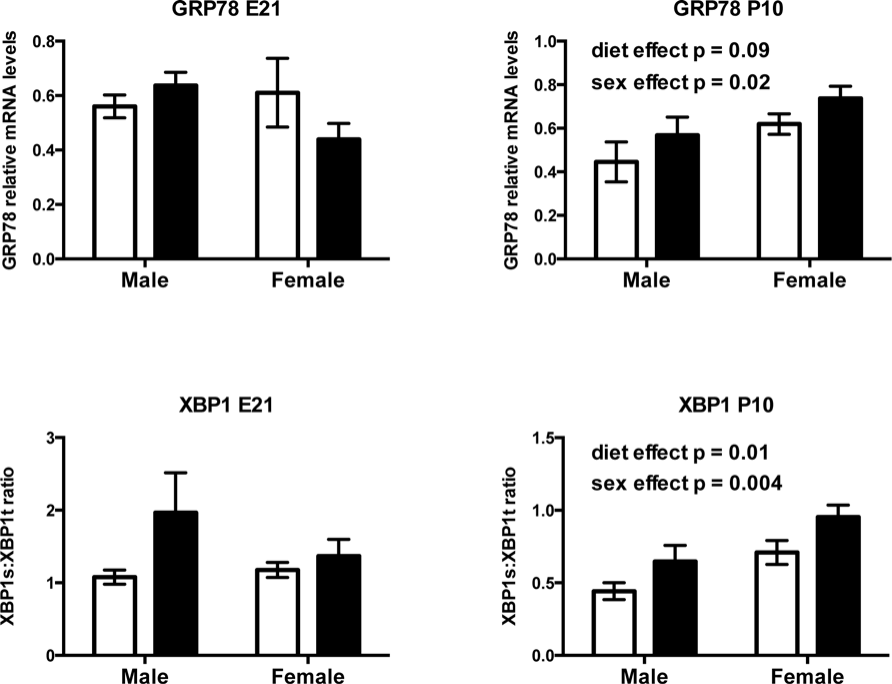
Prenatal fructose exposure induces hepatic ER stress in neonates at postnatal day 10. Offspring GRP78 and XBP1 mRNA levels at 2 timepoints. XBP1 is expressed as a ratio of spliced to total mRNA levels. Data are presented as means ± S.E.M. All mRNA levels are relative to the geometric mean of housekeeping genes. Two-Way ANOVA Main Effects are indicated in text where the 2 factors are maternal diet (fructose) and offspring sex. Control offspring are in open bars, fructose exposed offspring are in black bars. E21: embryonic day 21, P10: postnatal day 10. n = 6 per group per sex.

**Fig 9:**
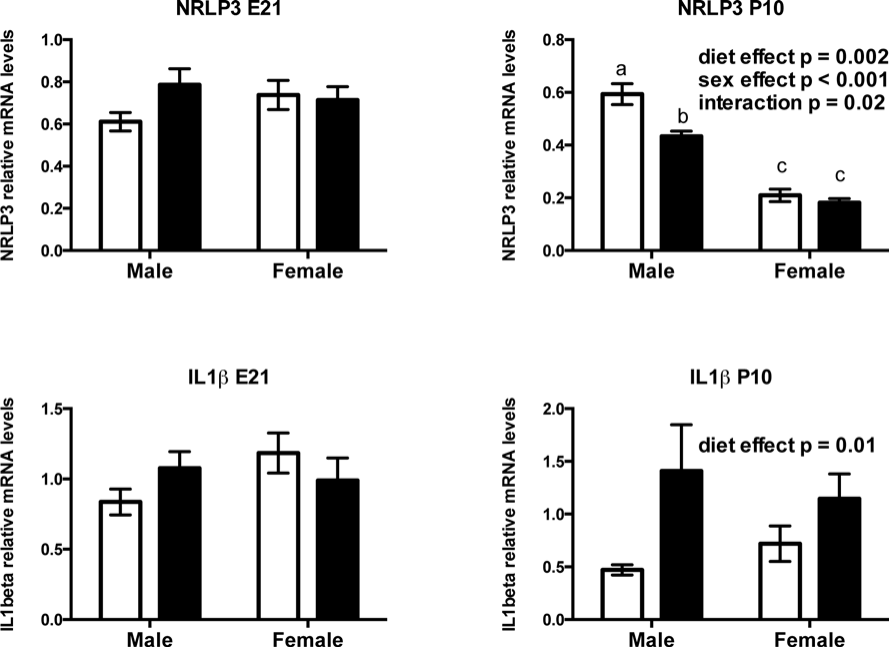
Maternal hepatic NLRP3 inflammasome and inflammatory signalling. Offspring NLRP3 inflammasome and IL-1β mRNA levels at 2 timepoints. Data are presented as means ± S.E.M. All mRNA levels are relative to the geometric mean of housekeeping genes. Two-Way ANOVA Main Effects are indicated in text where the 2 factors are maternal diet (fructose) and offspring sex. Tukey’s *post-hoc* analyses are indicated by letters, where bars with different letters indicate significance p < 0.05. Control offspring are in open bars, fructose exposed offspring are in black bars. E21: embryonic day 21, P10: postnatal day 10. n = 6 per group per sex. NLRP3: NOD-like receptor family, pyrin domain containing 3, IL1β: interleukin 1 beta.

Prenatal fructose exposure modestly elevated mRNA levels of the ER chaperone and signaling regulator GRP78 at P10 (p = 0.09) but not at E21 (Fig 8), and elevated a critical transcription factor, XBP1 in male and female neonatal livers at P10 (p = 0.01) in a sex dependent manner (p = 0.004). Prenatal fructose exposure induced an increase in XBP1s:XBP1t mRNA ratio at P10 (diet effect p = 0.01, sex effect p = 0.004) (Fig 8). In neonates, NRLP3 was decreased (main effect of diet p = 0.0023, effect of sex p <0.0001, interaction p = 0.022). Tukey’s post hoc analysis revealed that male, but not female, FR neonates had lower NRLP3 mRNA levels compared to controls (Fig 9). At P10 fructose exposed neonates had significantly elevated IL1β mRNA levels (main effect of diet p = 0.01, effect of sex p = 0.9, no interaction) regardless of sex (Fig 9). NFκB (RelA) and NFκBIA were similar between groups at both E21 and P10 timepoints (data not shown).

#### Prenatal fructose exposure alters expression of clock genes in neonates at P10

**Fig 10:**
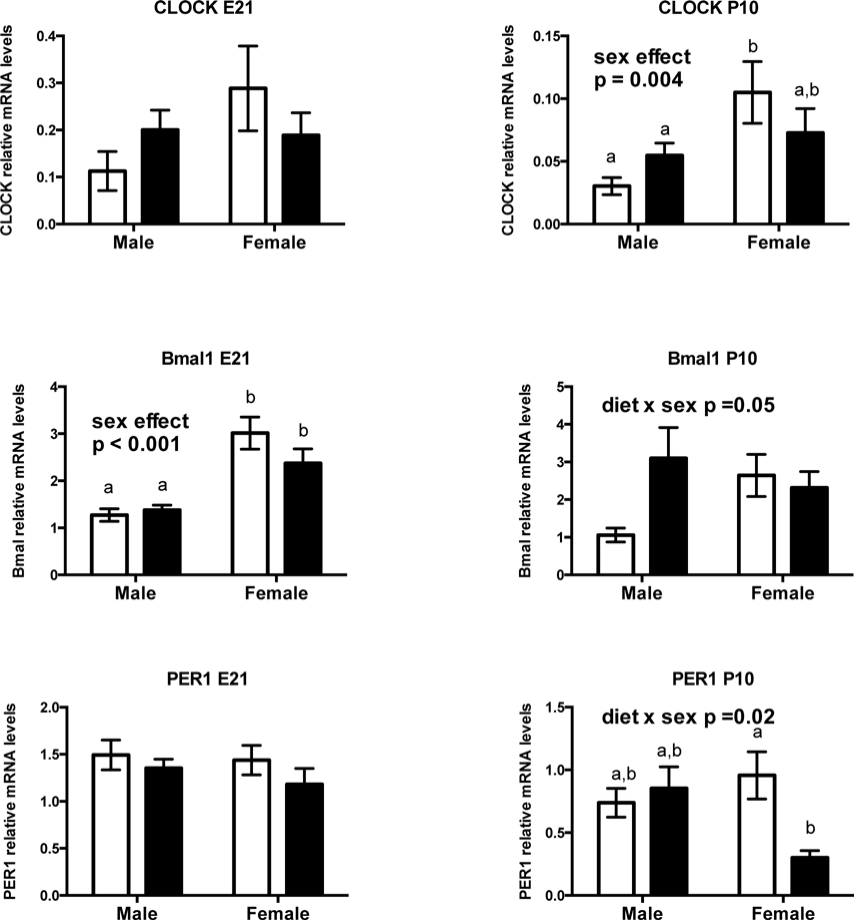
Prenatal fructose exposure alters neonatal hepatic clock gene expression levels in a sex dependent manner. Offspring CLOCK, Bmal1, and PER1 mRNA levels at 2 timepoints. Data are presented as means ± S.E.M. All mRNA levels are relative to the geometric mean of housekeeping genes. Two-Way ANOVA Main Effects are indicated in text where the 2 factors are maternal diet (fructose) and offspring sex. Tukey’s *post-hoc* analyses are indicated by letters, where bars with different letters indicate significance p < 0.05. Control offspring are in open bars, fructose exposed offspring are in black bars. E21: embryonic day 21, P10: postnatal day 10. n = 6 per group per sex. CLOCK: circadian Locomotor Output Cycles Kaput, Bmal1: also called Arntl Aryl hydrocarbon receptor nuclear translocator-like, PER1: Period 1.

Since changes in peripheral clock gene expression have been shown to exert effects on hepatic metabolic enzyme expression and function [30, 31],[32] we investigated whether prenatal fructose alters clock gene expression in mothers, fetuses and neonates. Maternal fructose intake did not alter mRNA expression levels of maternal hepatic Clock, Bmal, or Period genes (data not shown). Despite a significant effect of sex on Bmal1 mRNA levels at E21 (effect of sex p <0.001; females showing higher Bmal1 levels), we saw no effect of fructose exposure on fetal clock or Per1 genes (Fig 10). In neonates however, prenatal fructose exposure resulted in a significant interaction between diet and sex on Bmal1 (main effect of diet p = 0.1, sex effect p = 0.4, diet x sex interaction p = 0.05) and Per1 mRNA levels (main effect of diet p = 0.1, sex effect p = 0.3, diet x sex interaction p = 0.02), where fructose males had higher Bmal1 mRNA levels and FR females had lower PER1 mRNA levels (Fig 10).

## Discussion

We show that maternal fructose intake during pregnancy increased maternal hepatic ER stress, and resulted in hepatocellular injury and mRNA levels of genes that favour lipogenesis. Already at embryonic day 21, fetuses of mothers fed a high fructose diet displayed increased hepatic fructose transporter and reduced fructokinase mRNA levels and by 10 days of postnatal age, also exhibit hepatic lipid accumulation, hepatic ER stress and a suppression of genes regulating beta-oxidation. Critically, male and females show disparate gene expression profiles. These changes were not accompanied by changes in weight gain, food intake or serum glucose levels [21], suggesting that fructose-induced hepatic gene expression changes precede the evolution of outward obese phenotype and insulin resistance.

Although previous reports have indicated that prenatal fructose induces changes in hepatic fatty acid metabolism [15, 16], little is known regarding effects on the hepatic inflammasome in mothers and young offspring. Despite our recent data that maternal fructose induced increased circulating TNFα and IL1β [33], our data are consistent with others that hepatic IL1βmRNA is decreased in fructose fed mothers [33] and now show an associated decrease in NLRP3 mRNA levels. Although data in adult male rodents show that fructose exposure induced hyperuricemia, dyslipidemia leading to NLRP3 inflammasome activation [34, 35], it is likely that either sexually dimorphic effects (other studies have primarily been in males) or likely pregnancy-specific effects on fructose metabolism, as maternal metabolic adaptation to pregnancy results in different hepatic fuel utilization compared to adult male rodents.

### Prenatal fructose effects on maternal hepatic fructose, glucose and lipid metabolism

We have shown previously that fructose intake during pregnancy resulted in increased maternal plasma fructose and plasma insulin levels and increased maternal liver weight at E21 [21]. We also showed increased maternal plasma fructose levels, liver glycogen content and increased circulating triglycerides [21, 33]. Consistent with these observations, previous studies have documented maternal hyperinsulinaemia and increased liver weight following fructose consumption [36–38]. Despite an increase in total caloric intake in FR dams, maternal body weight gain during pregnancy or lactation was not different from CON dams; an observation consistent with other short-term studies of fructose consumption in animals and humans [14] That chronic consumption of lower doses of fructose can provoke hyperinsulinaemia and hepatic dysregulation has implications for women consuming substantial quantities of fructose or HFCS without experiencing weight gain or any outward phenotypic changes. As such our experimental paradigm may have utility as a model for maternal choleostasis and non-alcoholic fatty acid disease, particularly given the increases in liver ALT and AST enzyme concentrations. This is reinforced by our recent work showing that taurine supplementation can reverse some of the consequences of maternal fructose intake [33] and mirrors the hepatoprotective effects of taurine observed in the setting of cholestasis [39].

Not unexpectedly, maternal FR intake resulted in a significant increase in hepatic fructose dependent transporter GLUT5 (Slc2a5) mRNA levels. GLUT5 is the predominant fructose facilitated transporter [40]. Diets high in sucrose and fructose are known to upregulate GLUT5 and other isozymes in both animals and humans postnatally [41], and we now show similar changes in maternal liver both during pregnancy and lactation. Work by Alwahsh *et al*. has shown a correlation between GLUT5 mRNA and hepatic lipid accumulation in high fructose fed rats [42]. Similarly, work by Roncal-Jimenez *et al*. reports an upregulation of hepatic GLUT5 expression in high sucrose fed rats [43]. Recent work in a knockout mouse model has also reported a potential role for GLUT8 (Slc2a8) in hepatic fructose uptake as well as hepatic *de novo* lipogenesis but functionality has yet to be fully characterized [44].

FR mothers at P10 demonstrated increased levels of fructokinase, which likely contributed to elevated glycogen levels, consistent with our previous observation of increased maternal liver weight [21]. Fructokinase is stimulated by uric acid [45], and in a feed forward relationship, fructose metabolism results in increased uric acid synthesis [46, 47]. The increased production of uric acid is a result of fructokinase phosphorylating fructose to fructose-1-phosphate thus depleting cellular ATP and increasing the AMP/ATP ratio. In contrast to glucokinase, fructokinase is not downregulated by changes in cellular ATP depletion and thus ATP levels continue to fall as AMP rises, resulting in increased activity of AMP deaminase increasing degradation of AMP resulting in to uric acid synthesis [48]. Although it is likely that uric acid levels are increased in FR mothers in our study, we were unable to reliably measure uric acid. However, we have recently reported that maternal fructose intake increases maternal uric acid levels [33], consistent with other reports [49–51].

Our data support the notion that fructose ingestion leads to impairments in hepatic fatty acid metabolism and are consistent with other recent reports [14, 52]. FR mothers show increased levels of the transcription factor SREBP1c (at both E21 and P10, albeit modestly at P10), a key mediator in hepatic lipogenesis [53]. Previous work has shown that ER stress is a positive modulator of SREBP resulting in hepatic fatty acid accumulation through increased acetyl-CoA carboxylase (ACC) and fatty acid synthase (FAS) in non-pregnant female rats fed fructose [26]. We show that fructose intake during pregnancy and lactation has similar impacts in the mother on hepatic ER stress, increasing GRP78 during pregnancy at E21 and increasing XBP1s during lactation at P10.

Importantly, the target genes of SREBP1c extend beyond lipogenic genes. SERBP1c induces glucokinase, inhibits PEPCK and is mediated by insulin [53]. Our data are consistent with SREBP downregulating the rate limiting enzyme in gluconeogenesis, PEPCK, at both E21 and P10 although this effect appears not to be through SREBP’s action on SIRT1. FR mothers at P10 demonstrated an increase in PPARα, a key transcription factor regulating fatty acid oxidation. These data are in contrast to previous work showing that 60% fructose intake during pregnancy suppressed hepatic beta-oxidation and PPARα [54] consistent with a suppression of fatty acid utilization in favour of lipogenesis. We did not however see an increased in maternal liver triglyceride content in FR mothers, perhaps due to an increase in beta-oxidation. Despite the fact that we did not demonstrate increased hepatic triglyceride or increased levels of plasma NEFA in FR mothers, plasma levels of AST and ALT were significantly elevated in FR mothers, potentially suggestive of hepatocellular injurgy. This notion is consistent with results we have recently published showing that fructose induced hepatic steatosis in mothers [33].

Given the close relationship between hepatic inflammation and lipogenesis [55–57], we hypothesized that fructose induced lipogenesis would be mediated by an upregulation in the inflammasome signaling pathway. The NLRP3 inflammasome is a multi-protein complex that triggers the maturation of the pro-inflammatory cytokines IL-1β and IL-18 [58]. SREBP-1a has been shown to activate caspase-1 (pro-IL-1β) and stimulate IL-1β production and lipogenesis [59] and ER stress activated NLRP3 in pancreatic β-cells [60]. Importantly, the uric acid by-product of fructose metabolism can also directly stimulate the inflammasome [61, 62] and fructose-induced hyperuricemia and hyperlipidemia can activate the renal rNLRP3 inflammasome [63]. It is unclear why in FR mothers the NLRP3 inflammasome, including NFκB and IL-1β mRNA levels, are decreased, and whether FR mothers have suppressed immune function is unknown.

### Prenatal fructose effects on fetal and neonatal hepatic fructose, glucose and lipid metabolism

Fetuses of FR mothers demonstrated relatively few effects on hepatic gene expression levels at E21, with the exception of GLUT5 where mRNA levels were higher in FR female fetuses. Fetal hepatic fructokinase at E21, was decreased, but these differences were no longer evident by 10 days of postnatal life. As neonates FR males and females displayed sex-specific changes in genes that regulate hepatic fatty acid metabolism that were associated with increased markers of ER stress.

We demonstrate that in male neonates many key enzymes involved in hepatic beta oxidation including carnitine acyltransferase (CPT1a), and ketone metabolism (ACAT1,3) are suppressed after FR exposure. These changes are associated with increased mRNA levels of SREBP1c, suggesting that in male neonates hepatic fatty acid oxidation is decreased in favour of lipogenesis. These data, together with our observed increase in fatty acid transport (Slc27a3) are consistent with a modest increase in liver triglyceride levels. Previous work has shown that 10% fructose intake during pregnancy in the rat results in increased SREBP1c in the fetus [52]. We have previously shown that FR male neonates at P10 had higher plasma fructose levels compared to controls [21]. Whether changes in hepatic gene expression are the direct result of high circulating fructose levels or indirect, through exposure in the developmental period is unclear. Nevertheless, these data support the hypothesis that fructose induces gene expression changes in hepatic lipid regulation as early as 10 days after birth, and these changes are not evident in any changes in body weight [21]. Not unlike our observed decrease in mRNA levels of pro-inflammatory cytokines and NRLP3 inflammasome in the FR mothers, FR male neonates, but not females, also show decreased mRNA levels. Previous work suggests that offspring of nutrient restricted mothers show attenuated inflammatory response to LPS [64]. Whether offspring of FR mothers in this study go onto have compromised immune function later in life is unknown, and further studies investigating innate immunity in this model are warranted.

Neonatal FR females also demonstrated significant changes in hepatic levels of genes regulating fatty acid oxidation, but in distinctly different genes than those observed in FR male neonates. Overall, female FR neonates exhibited suppression of fewer genes responsible for beta-oxidation, and also exhibit suppressed levels of genes encoding fatty acid transport and binding. It is possible that this downregulation in genes encoding transport is compensatory as is not seen in male FR neonates. Interestingly, the one gene that was suppressed equally in both sexes was lipoprotein lipase (Lpl). Lpl is rate-limiting for the removal of serum triglycerides and is regulated by PPARα [65] [66]. Since fructose hepatic metabolism bypasses phosphofructokinase, a high flux of fructose to the liver may result in a marked increase in lipogenesis and produce very low density lipoproteins [67]. In females, our observed increase in liver triglyceride levels may be a result of reduced Lpl production due to reduced PPARα expression, and although male neonates also exhibited reduced LPL mRNA levels, this was not accompanied by reduced PPARα. This is consistent with others studies that show PPARα inhibition/mutations are associated with hypertriglyceridaemia and elevated hepatic triglycerides and PPARα agonists are effective in treating dyslipidaemia in type 2 diabetes patients and drugs that elevate hepatic lipoprotein lipase do so via PPARα activation [68].

Unique to FR female neonates was an increase in mRNA levels of the gene that encodes AMP kinase (Prkaa1). AMPK, a central metabolic controller, acts to increase cellular energy levels by inhibiting anabolic pathways, including fatty acid synthesis and stimulating energy catabolic pathways, such as fatty acid oxidation and glucose transport[69] AMPK inhibits ACC2 and SREBP1c resulting in a decrease in lipogenesis, and increasing in beta-oxidation in order to increase ATP levels [70]. However, the observed increase in AMPK mRNA levels in female neonates is discordant from SIRT1 and SREBP. Previous work has shown that SIRT1 induces AMPK phosphorylation and activation [71] and in turn AMPK mediates SIRT1 actions [72, 73]. Since AMPK activation requires phosphorylation at threonine 172 [74], it is possible that our observed decrease in mRNA levels of AMPK in FR female neonates does not translate into a reduction in AMPK phosphorylation. Indeed, recent work shows that in female offspring exposed to prenatal fructose, mRNA and total protein levels of AMPKα were unchanged, but levels of phosphorylated AMPKα protein were significantly lower in the liver and 4.6-fold higher in the hypothalamus. Consistent with this, protein levels of SIRT1, was significantly reduced in the fructose group of female offspring [38]. It is also possible that in the present study, FR female neonates have increased AMP/ATP ratios driving an upregulation of AMPK. Previous work in non-pregnant female rats fed fructose showed increased AMPK activity that was explained by increased expression of liver fructokinase in females but not males [75]. We were however unable to measure phosphorylated protein in the samples that were collected for the present study.

Disruptions of peripheral clock genes mediate impairments in hepatic metabolic regulation, where a down regulation of key components of clock transcriptional machinery is associated with hepatic insulin resistance and clock gene-mediated changes in SIRT1 expression [30]. It has also been shown that prenatal maternal nutritional manipulation reduces hepatic clock gene levels in offspring resulting in increased hepatic lipogenesis, before the onset of an obese phenotype [76]. We demonstrate that maternal FR intake results in modest changes in Bmal mRNA levels in a sex dependant manner. Our data are not dissimilar to a recent study demonstrating that offspring born to obese mothers, who were then challenged with a postweaning HF diet, showed decreased mRNA levels of clock core genes including CLOCK, Bmal, Per and Cry, as well as PPARα and SIRT1 [32]. In our study, it is possible that Period genes in the liver of female neonates may be facilitating a down regulation in SIRT1 and PPARα, thus imparting changes in hepatic beta-oxidation. It is tempting to speculate that clock-controlled genes are set up early in life and act as upstream mediators of hepatic metabolic activity. If so, then early life fructose exposure may impart changes in hepatic gene expression through clock-controlled mechanisms, but future studies are needed to determine expression of hepatic metabolic genes throughout the circadian cycle.

The reason for the differential responses to maternal FR by male and female offspring is unclear, but may reflect a difference in hepatic uptake and metabolism of fructose between the sexes. Previous studies have shown sex-specific differences in fructose responsiveness; Galipeau *et al*. showed that female rats were protected against fructose-induced changes in metabolism and blood pressure and a phenotype developed only after ovariectomy, thus implicating female sex hormones in the protection against fructose exposure [77]. These observations have been backed in part by human studies whereby associations between fructose and blood pressure were observed more frequently in girls than boys [78]. Similarly, sex-specific associations between the putative fructose transporter GLUT5 and uric acid levels have been reported [79] with effects more pronounced in females that males. Our results may suggest an effect of fetal estrogenicity on responsiveness to fructose exposure and therefore warrants further investigation. We have previously shown that male FR fetuses have elevated levels of the non-essential amino acid taurine compared to CON males [21]. Taurine is a potent hypoglycaemic compound, which augments insulin action and enhances glucose oxidation [80]. Taurine supplementation attenuates hypertension and improves insulin sensitivity in insulin resistant fructose-fed rats [81], so it may be that high plasma taurine leaves FR male fetuses relatively protected from the effects of fructose [21]. In this regard, a recent study by our group shows that maternal taurine supplementation ameliorates adverse metabolic sequelae in offspring following both a maternal obesogenic [82] and a fructose diet [33].

In conclusion, we present data that maternal fructose intake results in age- and sex-specific alterations in maternal, fetal and neonatal fatty acid metabolism. Importantly, these changes precede any gross changes in neonatal growth or weight changes, but are still characteristic of a loss of hepatic regulatory control of lipogenesis. In the context of the present study, it is important to recognise the potential differences in lipid metabolism between the rodent and human re translation of the experimental data. However, the rodent is similar to the human in that the primary site of *de novo* lipid synthesis is the liver whereas in other experimental species the primary site of synthesis is in the adipose tissue [83]. Whether these changes are associated with impaired hepatic inflammatory processes is still unclear, although suppression of the hepatic inflammasome in fructose fed mothers and male neonates points toward impaired immune sensing. These data further contribute to our understanding of effects of maternal fructose intake during critical periods of fetal and neonatal liver development and suggest that molecular changes are likely to occur very early in life, far earlier than the onset of an outward obesogenic phenotype.
